# Network pharmacology and experimental validation to elucidate the pharmacological mechanisms of OATF against kidney stones

**DOI:** 10.3389/fphar.2025.1575270

**Published:** 2025-05-13

**Authors:** Sisi Dai, Qi Fang, Hong-Yan Li, Rui Sun, Hui-Yong Zhang, Wei Wu

**Affiliations:** ^1^ School of Life Sciences, Zhuhai College of Science and Technology, Zhuhai, China; ^2^ College of Life Sciences, Jilin University, Changchun, China; ^3^ Key Laboratory of Ministry of Education for TCM Viscera-State Theory and Applications, Liaoning University of Traditional Chinese Medicine, Shenyang, China; ^4^ Department of Traditional Chinese Medicine, The Forth Affiliated Hospital of China Medical University, Shenyang, China; ^5^ School of Basic Medical Sciences, Peking University Health Science Center, Beijing, China

**Keywords:** Orthosiphon aristatus, kidney stone, apoptosis, network pharmacology, EGFR/PI3K/Akt

## Abstract

**Introduction:**

*Orthosiphon aristatus* (Blume) Miq. (OA) is widely used in folk medicine to treat kidney stones (KS). Its total flavonoids (OATF) are the primary active constituents responsible for its therapeutic effects. However, the exact mechanism of action (MOA) remains unclear. This study aimed to investigate the pharmacological activity of OATF against KS and elucidate its underlying MOA.

**Methods:**

Network pharmacology and molecular docking were utilized to predict the potential targets and pathways of OATF. An animal model of calcium oxalate crystal deposition was created using intraperitoneal injections of ethylene glycol (EG) and ammonium chloride (AC), alongside a model using human renal tubular epithelial cells (HK-2) induced by supersaturated oxalate (Ox) to investigate the pharmacological mechanisms of OATF against oxidative stress and apoptosis. The effects of OATF on crystal deposition and renal damage were assessed using hematoxylin-eosin (H&E) and periodic acid-Schiff (PAS) staining. Renal tubular damage and apoptosis were evaluated via TUNEL staining. The MOA was explored using Western blotting analyses.

**Results:**

Network pharmacological analysis identified the EGFR/PI3K/AKT pathway as a key mechanism in KS. *In vitro* experimental results demonstrated that OATF effectively protected HK-2 cells from oxidative stress, inhibited calcium oxalate crystal adhesion, and reduced apoptosis. *In vivo*, OATF significantly decreased serum creatinine (SCR), serum calcium (Ca), serum phosphorus (P), and blood urea nitrogen (BUN) levels in CaOx-induced Sprague-Dawley (SD) rats, indicating its protective effects against KS.

**Conclusion:**

OATF effectively inhibited kidney stone formation and mitigated renal injury by attenuating oxidative stress and apoptosis through activation of the EGFR/PI3K/AKT pathway. These findings highlight OATF’s therapeutic potential for KS management and provide a scientific basis for its traditional use in herbal medicine.

## 1 Introduction

Kidney stone (KS) is a prevalent disease affecting approximately 10%–15% of the global population and is the most common urological disorder (Yuan et al., 2023). Epidemiological studies indicate that the incidence and prevalence of kidney stones are increasing annually (Tundo et al., 2021; Hill et al., 2022). Renal stones are formed due to the crystallization and deposition of salts on the surface of renal epithelial cells, which leads to the formation of stones. KS can be classified into several types, including calcium oxalate, calcium phosphate, urate, magnesium ammonium phosphate, and cystine stones, in which calcium oxalate stones is the most common, accounting for over 80% of all cases (Daudon et al., 2016). The primary component of calcium oxalate stones is inorganic calcium oxalate crystals. In addition to causing pyelonephritis and renal injury due to urinary tract obstruction, the early deposition of calcium oxalate crystals can damage tubular epithelial cells and trigger inflammatory reactions (Ye et al., 2022). In severe cases, the progression of stone formation may result in renal failure (Wu et al., 2024). The clinical treatment of kidney stones primarily includes conservative drug therapy and surgical intervention. Drug therapy is mainly recommended for stones smaller than 6 mm in diameter, aiming to facilitate stone passage and alleviate renal colic using medications such as α-blockers, calcium channel antagonists, and other expulsive agents, as well as pain relievers like non-steroidal anti-inflammatory drugs (Al-Shawi et al., 2022). However, there is currently no effective pharmacological treatment for stones larger than 6 mm, which are primarily managed through minimally invasive surgery or extracorporeal shock wave lithotripsy (Druskin and Ziemba, 2016; Pavlov et al., 2016; Jiang et al., 2022). These approaches, however, are associated with significant side effects, high recurrence rates, and repeated procedures may result in impaired renal function (Kumar et al., 2020). Additionally, there is a lack of effective treatments to mitigate kidney damage caused by calcium oxalate crystal deposition (Gu et al., 2022). Consequently, there is an urgent need to identify novel therapeutic targets and develop new drugs to reduce the incidence of kidney stones and treat stone-induced kidney injury.

Increasing scientific research has revealed that natural active ingredients, such as polyphenolic compounds, flavonoid compounds, and others, play a key role in reducing the inflammation and oxidative stress associated with kidney stones. These active compounds exhibit antioxidant and anti-inflammatory properties that help mitigate oxidative stress and inflammatory responses, thereby reducing the damage caused by kidney stones (Sun et al., 2024). A key advantage of these remedies is their relatively low side-effect profile and safety, offering a gentler and potentially more effective treatment option for kidney stone patients. *Orthosiphon aristatus* (OA), also known with another name of kidney tea, is a plant of the genus *Orthosiphon* in the family which has been used for more than 1,000 years in China as a popular folk tea and traditional herbal medicine (Zhou et al., 2024a). OATF are bioactive constituents extracted from OA, mainly composed of flavonoids. Increasing pharmacological studies indicate that the anti-urolithic activity of flavonoids may be attributed to their diuretic, antioxidant, anti-inflammatory, antibacterial, and anti-apoptotic properties (Zargar et al., 2023). Additionally, flavonoids help protect and improve blood flow in the kidney’s small blood vessels (capillaries and arterioles), thereby safeguarding microcirculation (Cravotto et al., 2018). They also regulate the synthesis and expression of endogenous stone activators and inhibitors, maintain oxalate metabolism homeostasis, and reduce both the size and number of crystals (Chung et al., 2020; Zhu et al., 2024). Related studies showed that adenosine A(1) receptor antagonists could induce diuretic effects and promote sodium excretion, and seven methoxyflavonoids isolated from the methanol-water extract of kidney tea have been identified as active ligands for the adenosine A(1) receptor, demonstrating a positive correlation between their receptor affinities and diuretic activity. Furthermore, total flavonoids from kidney tea have been shown to alleviate oxidative stress and inflammation in kidney stone model mice by modulating the composition of omega-3 and omega-6 fatty acids in glycerophospholipids. This modulation significantly improved Ca^2+^ metabolism and alleviated the symptoms of renal stones while restoring the decline in renal function induced by stone formation (Chao et al., 2020).

Network pharmacology enables the integration of target molecules, biological functions, and bioactive compounds into complex interaction networks. This approach aligns closely with the inherent principles of traditional Chinese medicine (TCM) and enhances our understanding of the mechanisms underlying TCM’s therapeutic effects (Luo et al., 2020). Using a network pharmacology approach, we investigated the potential targets and pathways through which OATF may attenuate CaOx-induced kidney injury. To test our hypothesis, we conducted both *in vivo* and *in vitro* experiments.

## 2 Materials and methods

### 2.1 Experimental cells and animals

Human kidney tubular epithelial cell-2 (HK-2) cells were purchased from the Cell Bank of the Typical Cultures Preservation Committee, Chinese Academy of Sciences (Catalogue No. GNHu47). A total of 32 SPF-grade male SD rats, 6–8 weeks old and weighing 180–220 g, were provided by Liaoning Changsheng Biotechnology Co., Ltd. (License No.: SYXK (Liao) 2020-0001). The animal experiments were approved by the Animal Ethics Committee of Liaoning University of Traditional Chinese Medicine (Ethical Approval No.: 21000042024012).

### 2.2 Reagents and materials

*Orthosiphon aristatus* (Blume) Miq. was purchased from Kunming Xuanqing Biotechnology Co., Ltd. (Kunming, China). Creatinine (Cr) and phosphorus assay kits were purchased from Nanjing Jiancheng Bioengineering Research Institute (Nanjing, China). The Urea (BUN) assay kit, Oxalic acid detection kit, Superoxide Dismutase (SOD) assay kit, Malondialdehyde (MDA) assay kit, Calcium (Ca) assay kit, BCA protein assay kit, Hematoxylin-Eosin (HE) staining kit, and Periodic acid Schiff (PAS) staining kit were purchased from Beijing Suolaibao Technology Co., Ltd. (Beijing, China). Von Kossa dye, HE dye, anti-AKT antibody, anti-p-AKT antibody, anti-PI3K antibody, anti-p-PI3K antibody, and anti-EGFR antibody were obtained from Wuhan Servicebio Technology Co., Ltd. (Wuhan, China).

### 2.3 Preparation of OATF

*Orthosiphon aristatus* (1.5 Kg) was soaked in 5 L of 75% ethanol for 24 h, followed by two rounds of decoction, each lasting 2 h. The resulting extract was filtered, concentrated using a rotary evaporator, and dried at 60°C to obtain the kidney tea alcoholic extracts. A total of 500 g of silica polyamide resin (60–80 mesh) was pre-treated according to the manufacturer’s instructions. Then, 10 g of the kidney tea alcoholic extracts was dissolved in 50 mL of water and applied to the resin. The sample was washed with water at a flow rate of 10 mL/min, followed by elution with a 60% ethanol solution at the same flow rate. The ethanol eluate was collected, rotary evaporated to dryness, and the total flavonoids of kidney tea were weighed and recorded.

### 2.4 Analysis of OATF by network pharmacology

#### 2.4.1 Screening and target prediction of active ingredients in OATF

Since kidney tea was not searchable in the TCSMP database, we conducted a literature review spanning the past 30 years to identify active components of total flavonoids in kidney tea, resulting in the identification of 28 active compounds. We utilized the HERB and PubChem databases to obtain SMILES numbers and chemical structure formulas for these active ingredients. Subsequently, we predicted the target genes associated with these compounds using the Swiss Target Prediction database. Targets with a probability greater than 0.1 were identified through screening. The resulting targets were then used as predicted targets for the active ingredients of the OATF drug components, taking into account the findings obtained.

#### 2.4.2 Screening of potential targets for KS

We used terms such as “kidney stones,” “Renal calculus,” and “nephrolithiasis” to search in databases including GeneCards (https://www.genecards.org), Therapeutic Targets Database (TTD, http://bidd.nus.edu.sg/group/ttd/ttd.asp), Pharm GKB (https://www.pharmgkb.org/), Online Mendelian Inheritance in Man (OMIM, https://www.omim.org), and DrugBank (https//go.drugbank.com/). After eliminating duplicate targets, we obtained potential targets associated with kidney stones (KS). To determine the intersection between OATF and KS targets, we plotted a Venn diagram.

#### 2.4.3 The construction of the drug-active ingredient-target interaction network

We imported drug and disease targets into Venny 2.1 to identify intersecting genes, which were summarized in a Microsoft Excel table and then imported into Cytoscape 3.9.1 to construct a “drug-active ingredient-target” network model. This model visualized the relationships between traditional Chinese medicine (TCM) ingredients and their targets. In the network, nodes represent herbs, ingredients, and targets, while edges represent the relationships between them. The “degree” value was calculated based on the number of connections each node has.

#### 2.4.4 Construction of the protein-protein interaction network and screening of key targets

To clarify the functional interactions between the screened potential proteins, we constructed a protein-protein interaction (PPI) network using the STRING database (https://string-db.org/). The constructed PPI network was used for cross-targeting, with the species set to “*Homo sapiens*” and the protein interaction threshold set to “medium confidence” (0.400). The results were visualized using Cytoscape and analyzed with the Cytoscape-Centiscape 2.2 plug-in. Nodes with “degree” values greater than two times the median were used to filter out the core targets, ultimately leading to the identification of key targets for the treatment of kidney stone disease by OATF.

#### 2.4.5 KS-related target gene ontology and KEGG pathway enrichment analysis for OATF

To clarify the role of core intersecting genes of total flavonoids from kidney tea in kidney stone-related gene functions and signaling pathways, GO data for the intersecting targets were retrieved from the Metascape website. Bubble plots were then generated using Microbiotics’ online GO with pathway enrichment analysis, with criteria limited to P < 0.05, and species set to human. The top 10 cellular components (CC), molecular functions (MF), biological processes (BP), and the top 20 KEGG-ranked pathways in GO were sorted by ascending P value to create the bubble plots.

#### 2.4.6 Molecular docking

The 2D SDF structure of the main active ingredient was obtained through the PubChem platform, processed for energy minimization using ChemBio3D, the protein structure of the key target was obtained through the RSCB PDB (http://www.rcsb.org/) database, water and small molecule ligands were removed using PyMol, and after the AutoDockTools addition of hydrogen atoms and converted to PDBQT format for docking, docked and evaluated for binding capacity using AutoDock, visualised using Pymol software.

### 2.5 *In vitro* pharmacological validation

#### 2.5.1 Cell culture, groups, treatment

HK-2 cells, an immortalized proximal tubular epithelial cell line derived from normal adult human male kidneys, were obtained from the Cell Bank of the Chinese Academy of Sciences (Shanghai, China). The cells were cultured in DMEM medium supplemented with 10% FBS, 100 U/mL penicillin, and 100 µg/mL streptomycin, and incubated at 37°C in a humidified atmosphere containing 5% CO_2_. HK-2 cells were cultured to 40%–50% confluence, after which DMEM complete medium containing various concentrations of OATF was added. The cells were further incubated for 24 h to reach 70%–80% confluence. Subsequently, they were treated with either DMEM basal medium or 1.0 mM oxalic acid solution, as described previously (Su et al., 2024).

#### 2.5.2 Cell viability and crystalline adhesion assay

Cell viability was assessed using the Cell Counting Kit-8 (CCK-8, Biyuntian, China). HK-2 cells were inoculated at a density of 1 × 10^4^ cells per well in 96-well plates and cultured until 70%–80% confluence was reached. The cells were treated with OATF at concentrations of 5, 10, 25, 50, 75, 100, and 125 μg/mL for 24 h. After treatments, the medium was aspirated, and the cells were washed twice with PBS. Subsequently, 110 μL of CCK-8 working solution, which consisted of 10 μL of CCK-8 reagent and 100 μL of basal medium, was added to each well. The plates were then incubated at 37°C for 1 h. Finally, absorbance was measured at 450 nm using an enzyme-linked immunosorbent assay (ELISA) reader (EnSight, PerkinElmer, United States).

HK-2 cells in the logarithmic growth phase were inoculated into 6-well plates at a density of 1 × 10^5^ cells per well, with 2 mL of medium added to each well. After adhering for 24 h, complete medium containing OATF at concentrations of 10, 25, 50, and 100 μg/mL was added, and the cells were cultured for an additional 24 h. Subsequently, 2 mL of a 1.0 mM oxalic acid solution was added, and the cells were incubated for another 24 h. The results were observed and documented photographically using a fluorescence inverted microscope. Observations were repeated three times, and the data were analyzed using ImageJ software.

#### 2.5.3 Cellular biochemical examination

Cells were cultivated and collected following the aforementioned method. The cell count was performed, and the cells were processed according to the kit instructions. For every 5 million cells, 1 mL of the relevant biochemical index extract was added. The cells were then sonicated in an ice-water bath using a cell disruptor at 200 W for 3 s, with a 10-s interval, repeated 30 times. Following sonication, the cells were centrifuged at 4°C for 10 min at 8,000 rpm. The supernatant was collected and kept on ice for measurement. Biochemical indices, including MDA, SOD and GSH-PX, were measured according to the kit instructions.

#### 2.5.4 Reactive oxygen species (ROS) assay

Intracellular ROS levels were assessed using 2,7-dichlorodihydrofluorescein diacetate (DCFH-DA). Cells treated for the designated time periods were washed twice with phosphate-buffered saline (PBS), then incubated with 10 μM DCFH-DA diluted in serum-free medium at 37°C. ROS activity was subsequently measured by flow cytometry with excitation at 488 nm.

#### 2.5.5 Annexin V/propidium iodide (PI) flow cytometry

Cells were cultivated and collected following the method described above. The Binding Buffer was diluted 1:9 in deionized water (2 mL of 10x Binding Buffer plus 18 mL of deionized water). The cells were then re-suspended in 1x Binding Buffer, adjusting the concentration to 1–5 × 10^6^ cells/mL. A 100 µL aliquot of the cell suspension was placed in a 5 mL flow-through tube, followed by the addition of 5 µL of Annexin V/FITC. The mixture was incubated for 5 min at room temperature, protected from light. Subsequently, 5 µL of propidium iodide (PI) solution and 400 µL of PBS were added, and the flow assay was performed immediately. Results were analyzed using FlowJo software.

### 2.6 *In vivo* experimental validation

#### 2.6.1 Animal model and treatment

Male Sprague-Dawley (SD) rats (n = 32), aged 5 weeks and weighing 180–220 g, were purchased from Liaoning Changsheng Biotechnology Co., Ltd. (Liaoning, China). All animals were housed under standard laboratory conditions with *ad libitum* access to food and water. After 7 days of acclimatization, the rats were randomly divided into four groups (n = 8 per group): normal control (NC) group, model group 1 (vehicle), model group 2 (OATF low-dose), and model group 3 (OATF high-dose). Normal consumption of standard animal feed for all groups, 8 rats in the NC group were provided with drinking water *ad libitum*, while 24 rats in the model group received an aqueous solution containing 0.75% EG and 0.75% AC *ad libitum* for 2 weeks. After the modeling period, rats in the OATF low-dose group were administered 200 mg/kg/day of OATF (2 mL/rat) by gavage, and rats in the OATF high-dose group received 500 mg/kg/day of OATF (2 mL/rat) by gavage for two consecutive weeks. Rats in both the NC and vehicle groups were administered 2 mL/day of excipient by gavage throughout the treatment period. The excipient consisted of ethanol, polyethylene glycol 400, Tween 80, and 5% dextrose in a ratio of 3:6:1:20. All animal experiments were conducted in accordance with the ethical guidelines of the Laboratory Animal Ethics Committee of Liaoning University of Traditional Chinese Medicine.

#### 2.6.2 Blood, urine and kidney tissue sample collection

On the 28th day of the experiment, rats in each group were placed in metabolic cages for 24-h urine collection. The urine volume of each rat was recorded, and samples were centrifuged at 2,000 rpm for 10 min at a low temperature. The supernatant was collected, and concentrated hydrochloric acid was added to inhibit bacterial growth. Samples were then stored at −80°C for the analysis of urinary oxalic acid (UOx) and urinary calcium (UCa). Following urine collection, the animals were anesthetized, and blood samples were collected from the abdominal aorta. These samples were centrifuged at 3,500 rpm for 15 min at 4°C. The serum supernatant was aspirated and stored at −80°C for the determination of serum creatinine (SCR), blood urea nitrogen (BUN), serum calcium (SCa), and serum phosphorus (SP). The kidneys were excised bilaterally, cleared of peritoneum and fat, and weighed to determine the kidney-to-body weight ratio. The left kidney of SD rats was dissected along the coronal plane, immediately immersed in 4% paraformaldehyde, and then embedded in paraffin for hematoxylin and eosin (H&E) staining, Von Kossa staining, and TUNEL staining. The right kidney was rapidly frozen in liquid nitrogen and stored at −80°C. A portion of the homogenate was used to measure tissue levels of malondialdehyde (MDA) and superoxide dismutase (SOD), and another portion was reserved for protein extraction.

#### 2.6.3 Determination of UOx, UCa, SCR, BUN, SCa and SP

UOx, UCa, SCa, and BUN levels were determined by colorimetric analysis using commercially available kits (Beijing Solarbio Biotechnology Co., Ltd., China) according to the manufacturer’s instructions. SCR and SP levels were measured using colorimetric kits from Nanjing Jiancheng Biotechnology Co., Ltd., China, as per the manufacturer’s protocols. UOx levels were quantified by sulfosalicylic acid complexation; UCa and SCa levels were determined using the o-cresolphthalein complexone colorimetric method; SP levels were assessed with the phosphomolybdic acid colorimetric method; SCR levels were measured via the sarcosine oxidase reaction method; and serum urea nitrogen was determined using the urease method.

#### 2.6.4 Determination of SOD and MDA

The levels of SOD and MDA were determined using commercially available kits (Beijing Solebo Biotechnology Co., Ltd., China) according to the manufacturer’s instructions. SOD levels were measured by the nitroblue tetrazolium (NBT) photoreduction method, while MDA levels were assessed using the thiobarbituric acid (TBA) method.

#### 2.6.5 Morphologic analysis and histologic scoring

PAS and H&E staining methods are commonly used to evaluate the extent of damage to renal tubules (Yang et al., 2020; Jiang et al., 2021; Liu et al., 2024). Kidney injury was assessed through morphometric analysis based on the following criteria from previous studies (Kumar et al., 2022): no injury (score 0), tubular dilatation and loss of the brush border (score 1), tubular epithelial cell vacuolization (score 2), apical blebbing (score 3), and epithelial cell sloughing with granular casts (score 4). The severity of kidney injury was quantified using the formula:

$$kidney\;injury\;score=\frac{\Sigma (Number\;of\;tubules\;with\;a\;score>0\times corresponding\;score}{Total\;numeber\;of\;tubles\;examined}$$

The renal cortex was evaluated in each section by examining ten high-power fields under 100× magnification.

#### 2.6.6 TUNEL staining

Renal apoptosis in paraffin-embedded left kidney tissues, sectioned to 4 mm thickness, was assessed using a TUNEL assay kit according to the manufacturer’s instructions. TUNEL-positive cells were counted in four randomly selected fields of view (400 × magnification) using a fluorescence microscope. The rate of apoptotic cells was analyzed with ImageJ software.

#### 2.6.7 Western blot analysis

Proteins were extracted from kidney tissue using RIPA buffer (Servicebio Co., Ltd., Wuhan, China). The extracted proteins were denatured by boiling in a metal bath at 100°C for 10 min. Total protein concentration was determined using a BCA protein assay kit (Servicebio Co., Ltd., Wuhan, China). Equal amounts of protein (20 μg) were separated by 10% sodium dodecyl sulfate-polyacrylamide gel electrophoresis (SDS-PAGE) and transferred to PVDF membranes (Servicebio Co., Ltd., Wuhan, China) via electrophoresis. The membranes were blocked in 5% skimmed milk dissolved in tris-buffered saline with Tween 20 (TBST) for 2 h at room temperature, followed by overnight incubation at 4°C with the following primary antibodies: anti-ACTIN (1:5,000), anti-AKT (1:1,000), anti-p-AKT (1:1,000), anti-PI3K (1:1,000), anti-p-PI3K (1:1,000), and anti-EGFR (1:1,000). Subsequently, the membranes were incubated with HRP-conjugated secondary antibodies (anti-goat/rabbit). The blots were visualized using an enhanced chemiluminescence (ECL) system, and the molecular weight and optical density of the target bands were analyzed using AIWBwell™ software.

### 2.7 Statistical analysis

All data are expressed as mean ± standard deviation (SD). Statistical analyses and graph generation were performed using GraphPad Prism nine software. All *in vitro* experiments were conducted at least three times. Comparisons between two groups were made using two-sided Student’s t-test, while one-way analysis of variance (ANOVA) was used to assess differences among multiple groups. P values less than 0.05 were considered statistically significant.

## 3 Results

### 3.1 Network pharmacology analysis

**FIGURE 1:**
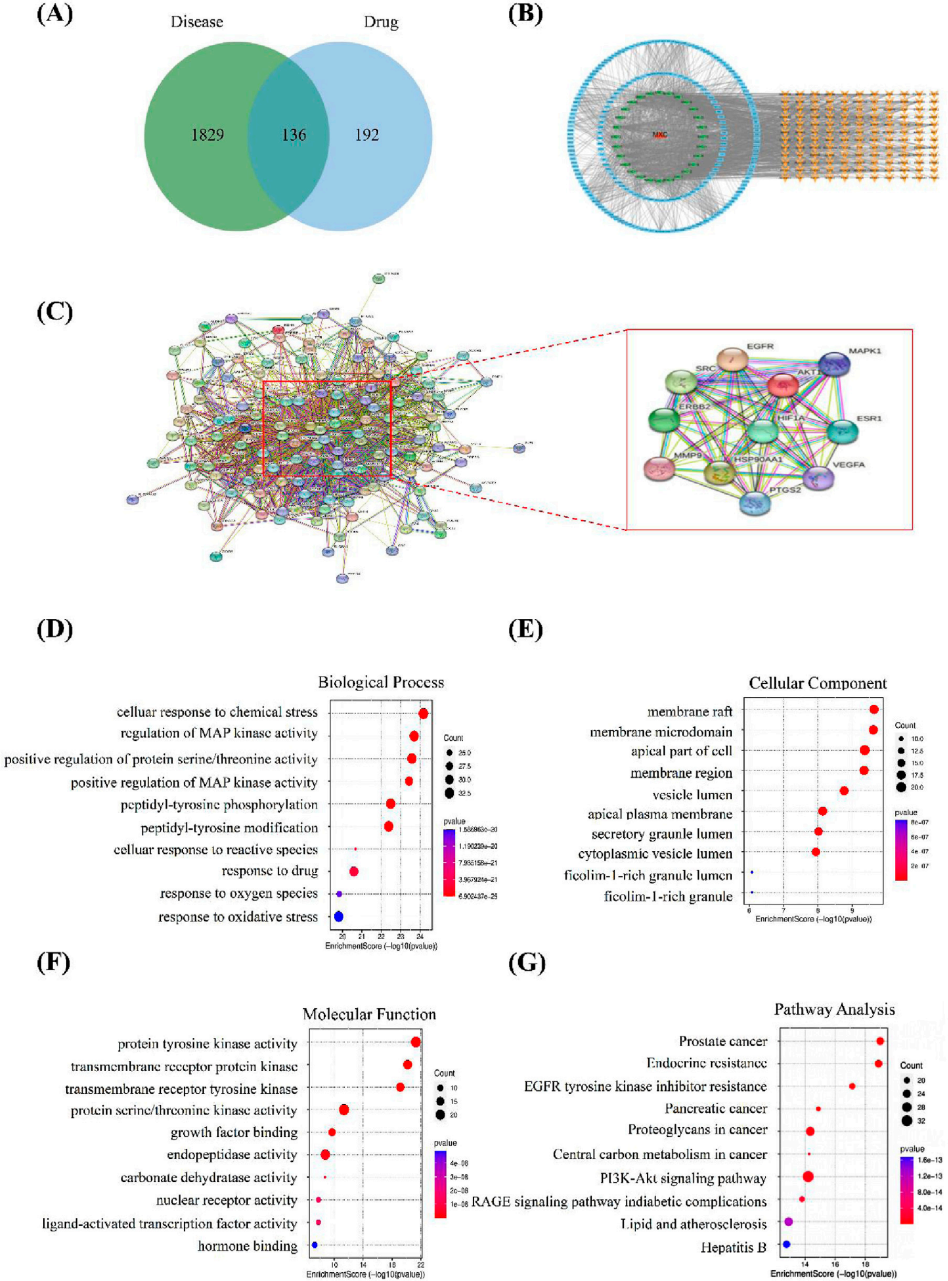
Network pharmacology analysis. **(A)** Venn diagram illustrating the overlapping genes targeted by both OATF and KS. **(B)** OATF active ingredient-KS target network map. **(C)** PPI network of targets generated using STRING 11.0. **(D)** Top 10 biological processes in Gene Ontology of 136 targets. **(E)** Top 10 cellular components in Gene Ontology of 136 targets. **(F)** Top 10 molecular functions in Gene Ontologyof 136 targets. **(G)** Top 10 pathways in the Kyoto Encyclopedia of Genes and Genomes (KEGG) of 136 potential targets. The abscissa indicates gene proportion, the ordinate shows pathway name, bubble size represents the number of targets in the pathway and colour indicates p-value.

We used the HERB and PubChem databases to obtain SMILES codes or chemical structures of active components. Subsequently, target names were annotated using the UniProt database, and target genes associated with these components were predicted through Swiss Target Prediction. Targets with a probability greater than 0.1 were selected (Table 1). A total of 1,046 KS-related target genes were identified after removing duplicates. The 328 targets of OATF’s active components and 1,965 KS-related targets were imported into the Venn 2.1.0 platform, identifying 136 potential targets for OATF in KS treatment, as shown in the Venn diagram (Figure 1A). Using Cytoscape 3.7.2, we constructed a drug-compound-target network consisting of 380 nodes and 2,174 edges. In this network, red nodes represent renal tea, green nodes represent OATF, blue nodes denote the active targets of each compound in OATF, and orange nodes signify KS-related targets (Figure 1B). Additionally, a protein-protein interaction (PPI) network for these shared targets was generated using the STRING platform (Figure 1C). Core targets were identified using the CentiScape 2.2 plugin in Cytoscape by calculating degree centrality (DC), betweenness centrality (BC), and closeness centrality (CC) values. A total of 11 core targets were screened, as shown in Table 1.

**TABLE 1:** Basic topological properties of core regulatory genes.

Geen symbol	Average shortest path length	Degree	Betweenness	Closeness	Neighborhood connectivity
AKT1	1.30075188	186	1,773.941162	0.005780347	31.91397849
EGFR	1.345864662	178	1,309.855118	0.005586592	32.51685393
HIF1A	1.428571429	158	749.8786882	0.005263158	34.94936709
BCL2	1.413533835	156	740.7606199	0.005319149	35.42307692
ESR1	1.413533835	156	1,016.205793	0.005319149	35.03846154
SRC	1.458646617	154	1,122.375051	0.005154639	34.31168831
HSP90AA1	1.473684211	144	791.3808042	0.005102041	35.83333333
MMP9	1.481203008	144	709.1360207	0.005076142	35.13888889
ERBB2	1.503759398	140	407.5218589	0.00500000	36.5000000
PPARG	1.541353383	124	810.1469517	0.004878049	37.27419355

To elucidate the pharmacological mechanism of OATF in treating KS, core targets with the highest degree values were subjected to Gene Ontology (GO) functional annotation and KEGG pathway enrichment analyses. The GO analysis identified 6,273 enriched entries, including 4,966 related to biological processes (BP), 844 related to molecular functions (MF), and 463 associated with cellular components (CC). The BP analysis indicated involvement in processes such as cell response to chemical stress, regulation of MAP kinase activity, and positive regulation of protein/threonine kinase activity (Figure 1D). The CC analysis highlighted structures such as membrane rafts, membrane microdomains, the apical part of cells, and membrane regions (Figure 1E). The MF analysis revealed associations with protein tyrosine kinase activity, transmembrane receptor protein kinase activity, and transmembrane receptor protein tyrosine kinase activity (Figure 1F). KEGG pathway enrichment analysis identified 244 pathways, with significant associations observed for the EGFR and PI3K/AKT signaling pathways (Figure 1G). These results provide insights into the potential mechanisms of OATF in the treatment of KS.

### 3.2 Molecular docking study

**FIGURE 2:**
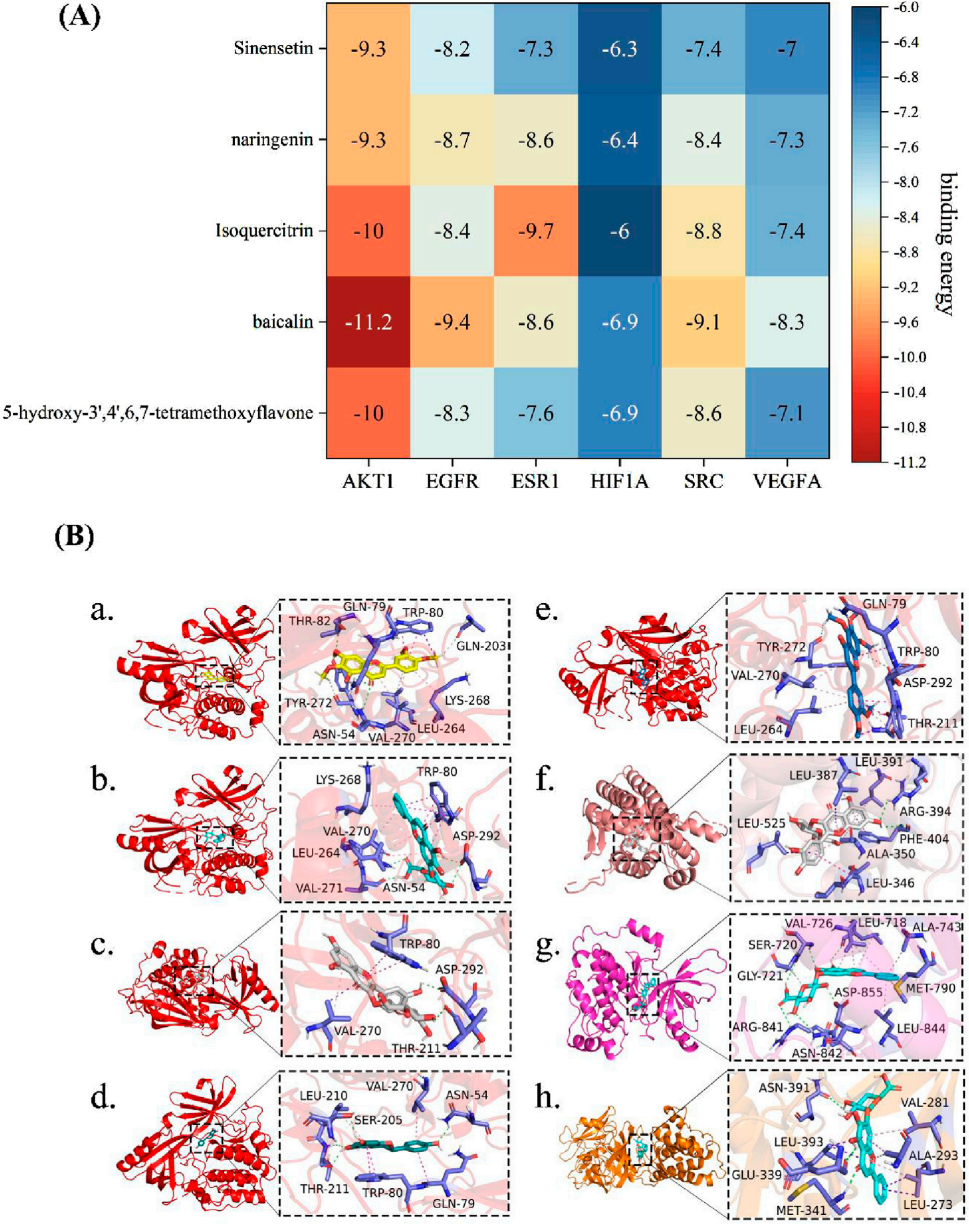
Molecular docking between main ingredients of OATF and KS-related proteins. **(A)** Heatmap of binding energy (kcal/mol) of core targets and active ingredients of OATF; **(B-a)** binding poses of 5-hydroxy-3′,4′,6,7 tetramethoxyflavone complexed with AKT1, affinity = −10 kcal/mol; **(B-b)** binding poses of baicalin complexed with AKT1, affinity = −11.2 kcal/mol; **(B-c)** binding poses of Isoquercitrin complexed with AKT1, affinity = −10 kcal/mol; **(B-d)** binding poses of Isoquercitrin complexed with AKT1, affinity = −9.3 kcal/mol **(B-e)** binding poses of Sinensetin complexed with AKT1, affinity = −9.3 kcal/mol; **(B-f)** binding poses of baicalin complexed with EGFR, affinity = −9.4 kcal/mol; **(B-g)** binding poses of Isoquercitrin complexed with EGSR1, affinity = −9.7 kcal/mol; **(B-h)** binding poses of baicalin complexed with SRC, affinity = −9.4 kcal/mol.

To investigate the interactions between the core active components of OATF (5-hydroxy-3′,4′,6,7-tetramethoxyflavone, baicalin, iso-quercetin, (2S)-naringenin) and four key disease-related genes (AKT1, EGFR, ESR1, SRC), we conducted a molecular docking analysis. Correlation studies indicated that a ligand could automatically bind to a receptor when the docking score was <0 kcal/mol, with optimal docking observed at scores < −5.0 kcal/mol (Li et al., 2020). The docking results showed that the binding energies of OATF’s core active components were low for all four target genes (Figure 2A), suggesting a strong affinity and spontaneous binding between them. The best binding energy observed was −11.2 kcal/mol, while the worst was −6 kcal/mol. These results indicate that the core components in OATF can bind effectively to these four critical drug targets. The findings are visualized in Figure 2B.

### 3.3 OATF inhibits calcium oxalate crystal adhesion and mitigates oxidative stress in HK-2 cells

**FIGURE 3:**
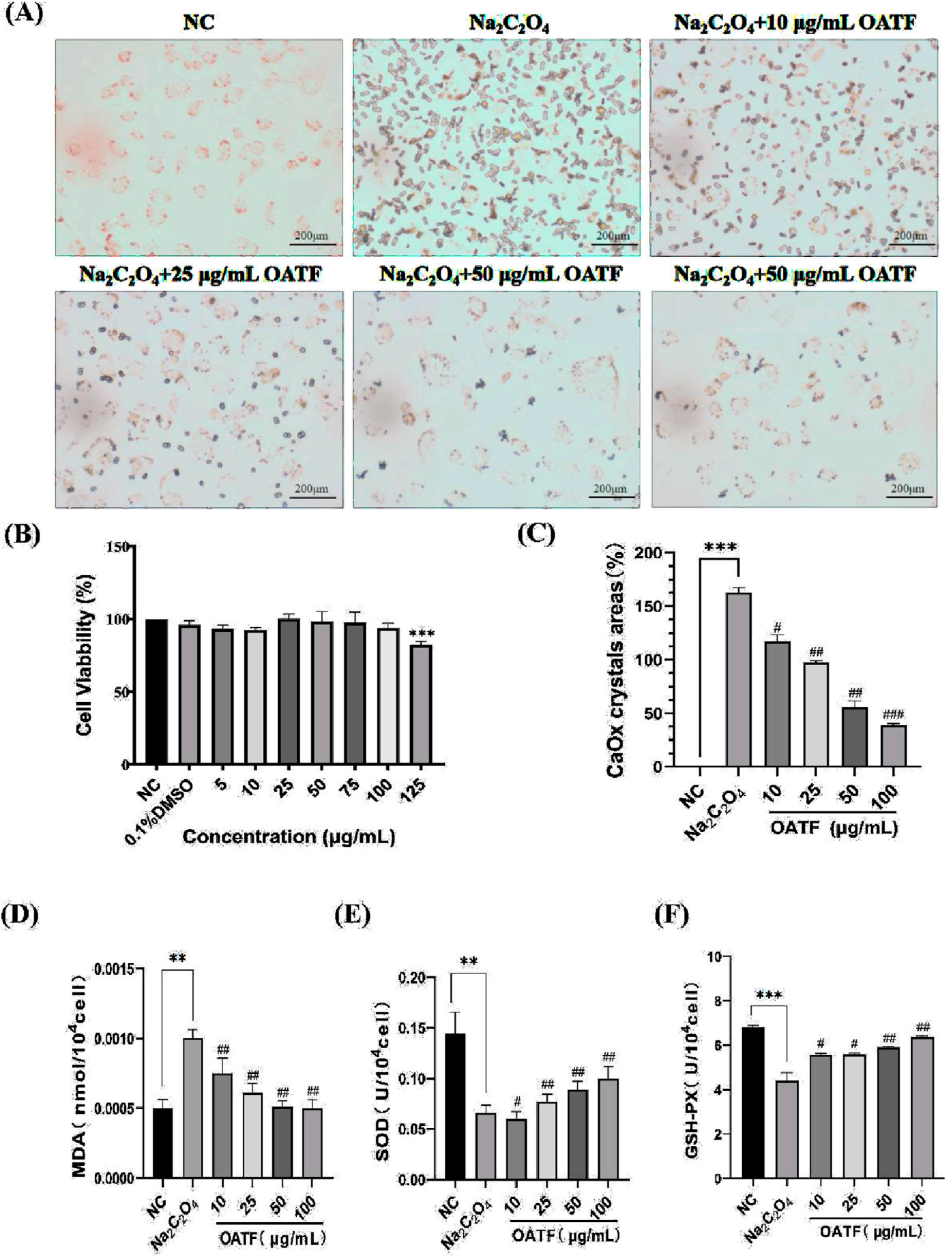
Effect of OATF on cell viability, calcium oxalate crystal formation, and oxidative stress in HK-2 cells. **(A)** Representative images of CaOx crystal adhesion observed under an inverted microscope (40×, Scale bar = 200 μm). **(B)** CCK-8 assay results showing the cytotoxicity of different OATF concentrations on HK-2 cells. **(C)** Quantification of crystal adhesion in HK-2 cells following OATF pretreatment at various concentrations. **(D)** SOD activity in the kidneys of rats. **(E)** MDA content in the kidneys of rats. **(F)** GSH-PX activity in the kidneys of rats. Data are presented as mean ± SD. ***p < 0.001 vs. the NC group; ^#^ *p* < 0.05, ^##^ *p* < 0.01, ^###^ *p* < 0.001 vs. the model group.

The cytotoxicity of OATF on HK-2 cells was evaluated using the CCK-8 assay, which confirmed that OATF (5–100 μg/mL) promoted HK-2 cell growth without cytotoxic effects (Figure 3B). To assess its impact on crystal adhesion, HK-2 cells were treated with Na_2_C_2_O_4_ to induce calcium oxalate (CaOx) crystal formation. The model group exhibited significantly increased crystal attachment compared to the normal control (NC) group, whereas OATF pretreatment (10, 25, 50, 100 μg/mL) reduced crystal adhesion in a concentration-dependent manner (Figure 3A). The crystal area was quantified using ImageJ, confirming the inhibitory effect of OATF on CaOx adhesion (Figure 3C).

Furthermore, oxidative stress markers were measured to evaluate the protective effect of OATF (Wang et al., 2022). As shown in Figures 3D–F, MDA levels were significantly elevated in the model group (*p* < 0.01), indicating enhanced lipid peroxidation. OATF pretreatment significantly reduced MDA levels in a dose-dependent manner (*p* < 0.01), mitigating oxidative stress induced by Na_2_C_2_O_4_. In addition, OATF enhanced the activities of SOD and GSH-PX, suggesting its potent antioxidant properties in preventing oxidative damage during CaOx crystal formation.

### 3.4 OATF decreases reactive oxygen species levels in HK-2 cells

**FIGURE 4:**
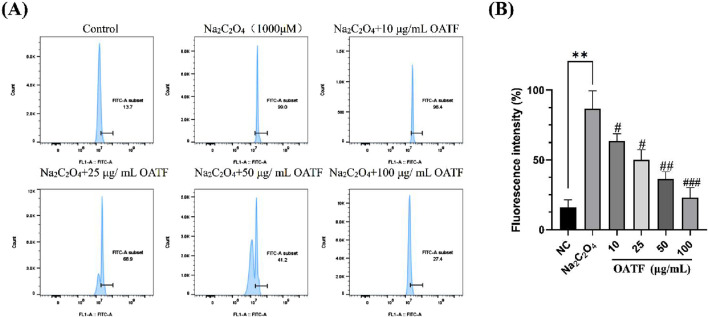
Effects of OATF on sodium oxalate-induced reactive oxygen species (ROS) in an HK-2 cell model. **(A)** Flow cytometric detection of ROS levels. **(B)** Statistical analysis of fluorescence intensity. ^**^ *p* < 0.01 vs. The NC group, ^#^ *p* < 0.05 vs. the model group, ^##^ *p* < 0.01 vs. the model group. ^###^ *p* < 0.001 vs. the model group.

DCFH is non-fluorescent, but in the presence of intracellular reactive oxygen species (ROS), it generates fluorescent DCF, allowing for the determination of intracellular ROS levels by measuring the fluorescence intensity of DCF. The results are shown in Figure 4. Compared to the control group, the ROS level in the model group significantly increased after treatment with 50 μg/mL Na_2_C_2_O_4_, indicating that Na_2_C_2_O_4_ treatment led to elevated ROS levels in the cells. In contrast, the ROS levels in HK-2 cells pre-treated with 10, 25, 50, and 100 μg/mL of OATF decreased compared to the model group.

### 3.5 OATF decreases apoptosis in HK-2 cells

**FIGURE 5:**
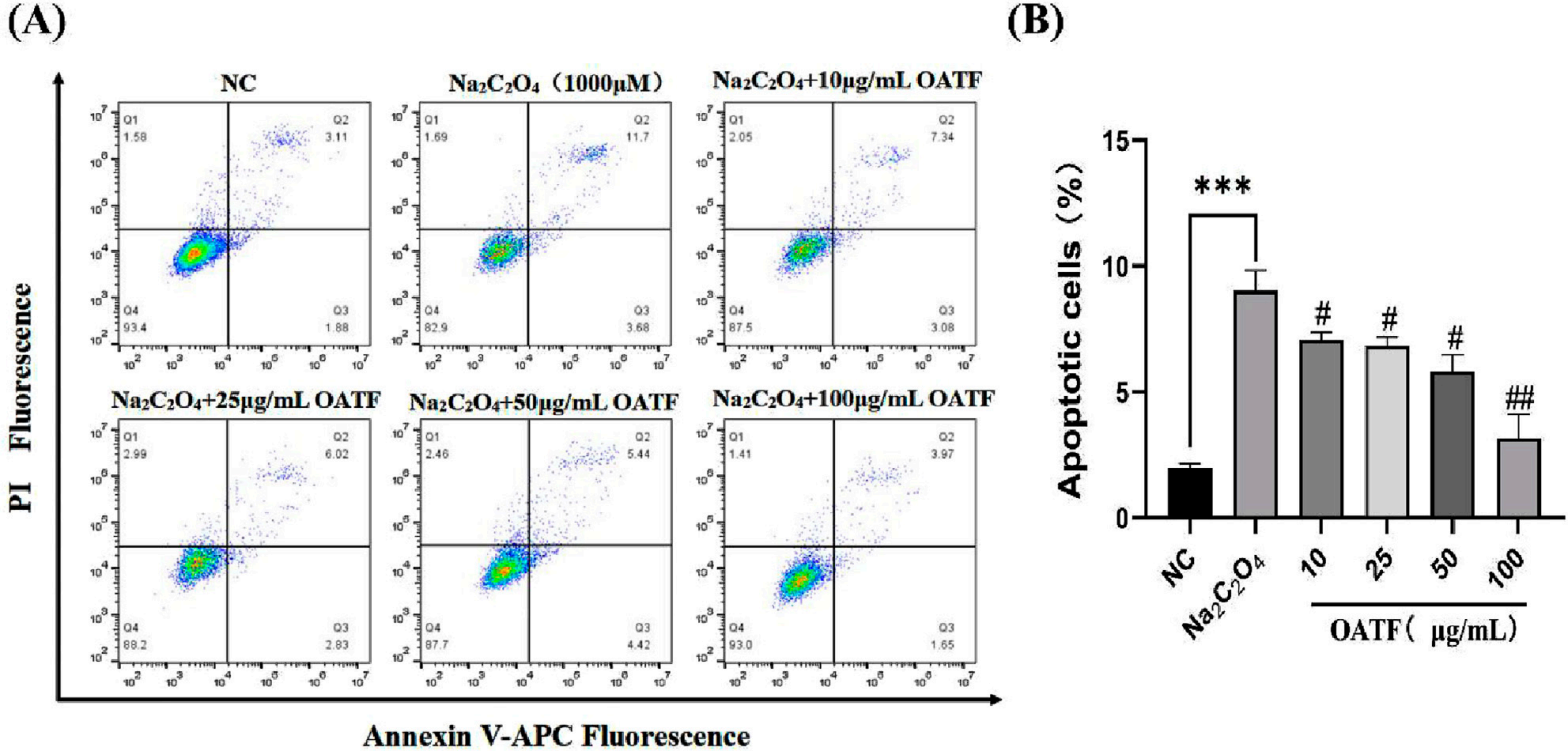
Levels of apoptosis induced by total flavonoids from renal tea in a sodium oxalate-induced HK-2 cell model. **(A)** Apoptosis levels detected by flow cytometry. **(B)** Statistical analysis of apoptosis rates in HK-2 cells across different groups. ^***^ *p* < 0.001 vs. The NC group, ^#^ *p* < 0.05 vs. the model group, ^##^ *p* < 0.01 vs. the model group.

The results are shown in Figure 5. Compared to the NC group, the apoptosis rate significantly increased to 9.03% ± 0.64% in the model group, indicating that Na_2_C_2_O_4_ treatment induced apoptosis. Treatment with different concentrations of OATF (10, 25, 50, and 100 μg/mL) decreased the apoptosis rate by 21.64%, 24.07%, 35.53%, and 65.33%, respectively, in a dose-dependent manner, all of which were statistically significant compared to the NC group. These results indicate that 24-h pretreatment with OATF effectively inhibits the level of apoptosis induced by Na_2_C_2_O_4_ injury.

### 3.6 OATF ameliorates EG + AC-induced renal dysfunction and oxidative stress in rats

**FIGURE 6:**
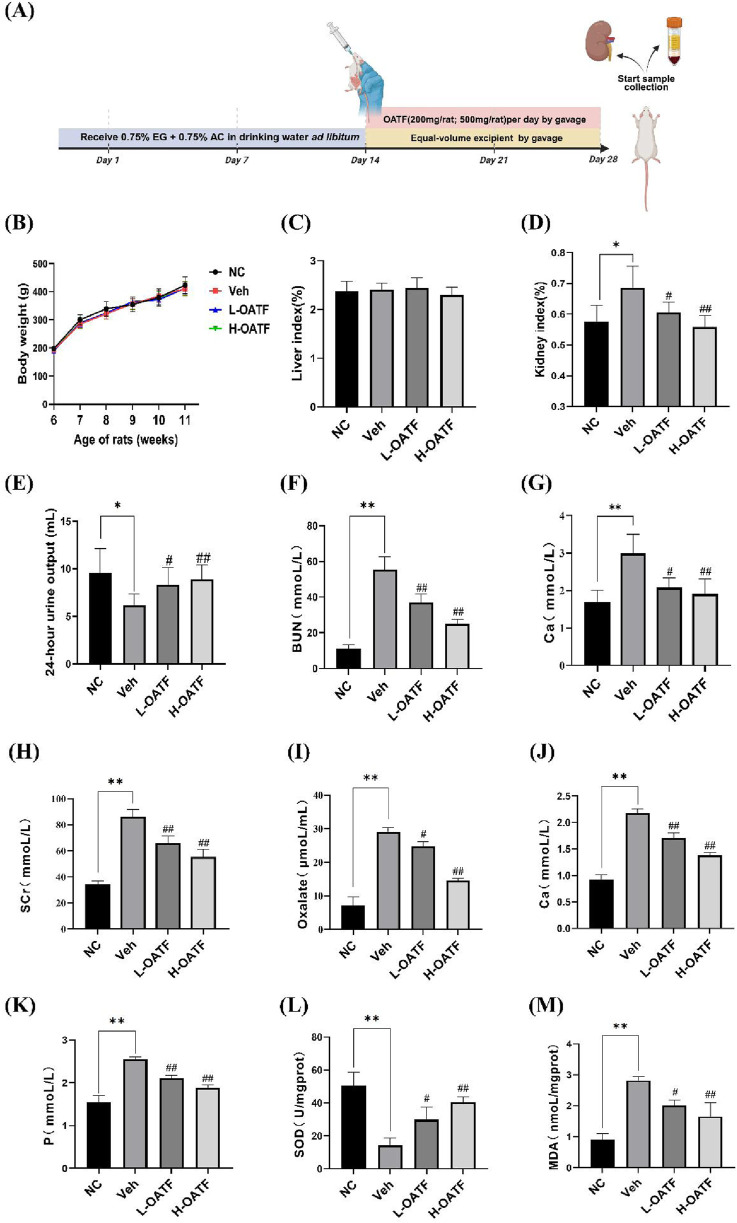
OATF ameliorates EG + AC-induced renal dysfunction and oxidative stress in rats. **(A)** Schematic diagram illustrating the experimental timeline (created with BioRender.com). **(B)** Changes in body weight during the modeling period. **(C)** Liver-to-body mass index. **(D)** Kidney-to-body mass index. **(E)** 24-h urine volume. **(F)** Urinary oxalate concentration. **(G)** Urinary calcium concentration. **(H)** Serum creatinine concentration. **(I)** Blood urea nitrogen concentration. **(J)** Serum calcium concentration. **(K)** Serum phosphorus concentration. **(L)** Renal SOD activity. **(M)** Renal MDA content. Data are presented as mean ± SD (n = 3 per group). ^**^ *P* < 0.01 vs. the NC group, ^#^ *P* < 0.05, ^##^ *P* < 0.01 vs. the Veh group.

To evaluate the role of OATF in improving renal function *in vivo*, we established a rat kidney stone model by administering ethylene glycol and ammonium chloride, followed by oral gavage of OATF (Figure 6A). Weekly body weight measurements showed no significant differences between groups (*P* > 0.05) (Figure 6B), and no significant changes were observed in the liver-to-body mass index (*P* > 0.05) (Figure 6C), indicating that OATF administration was safe. In contrast, the kidney-to-body mass index was significantly elevated in the model group (*P* < 0.05), while 24-h urine output was significantly reduced (*P* < 0.05). Notably, OATF administration significantly decreased the kidney-to-body mass index and increased 24-h urine output (Figures 6D,E). We further investigated whether OATF inhibited oxalate and calcium crystal formation in the animal model. Compared to the NC group, urinary oxalate and calcium levels were significantly elevated in the model group (*P* < 0.05). Concurrent treatment with OATF significantly reduced urinary oxalate and calcium levels (*P* < 0.05) (Figures 6F,G). Additionally, we measured serum levels of SCr, BUN, Ca, and P to assess the effects of OATF on renal function and serum biochemical indices. Rats in the model group exhibited significantly higher levels of SCr, BUN, Ca, and P compared to the NC group (*P* < 0.05). Simultaneous treatment with OATF resulted in a significant reduction in serum SCr, BUN, Ca, and P levels (*P* < 0.05) (Figures 6H–K).

Given the critical role of oxidative stress in hyperoxaluria-induced renal injury (Park et al., 2020; Khan et al., 2021; Li et al., 2021), we further investigated the antioxidant properties of OATF. The model group exhibited significantly increased renal MDA levels and decreased SOD activity compared to the NC group (*P* < 0.05). Notably, OATF treatment significantly reduced renal MDA levels while enhancing SOD activity (*P* < 0.05) (Figures 6L,M), suggesting that OATF mitigates oxidative stress and protects against kidney stone formation.

### 3.7 OATF attenuates renal pathological changes and apoptosis in rats

**FIGURE 7:**
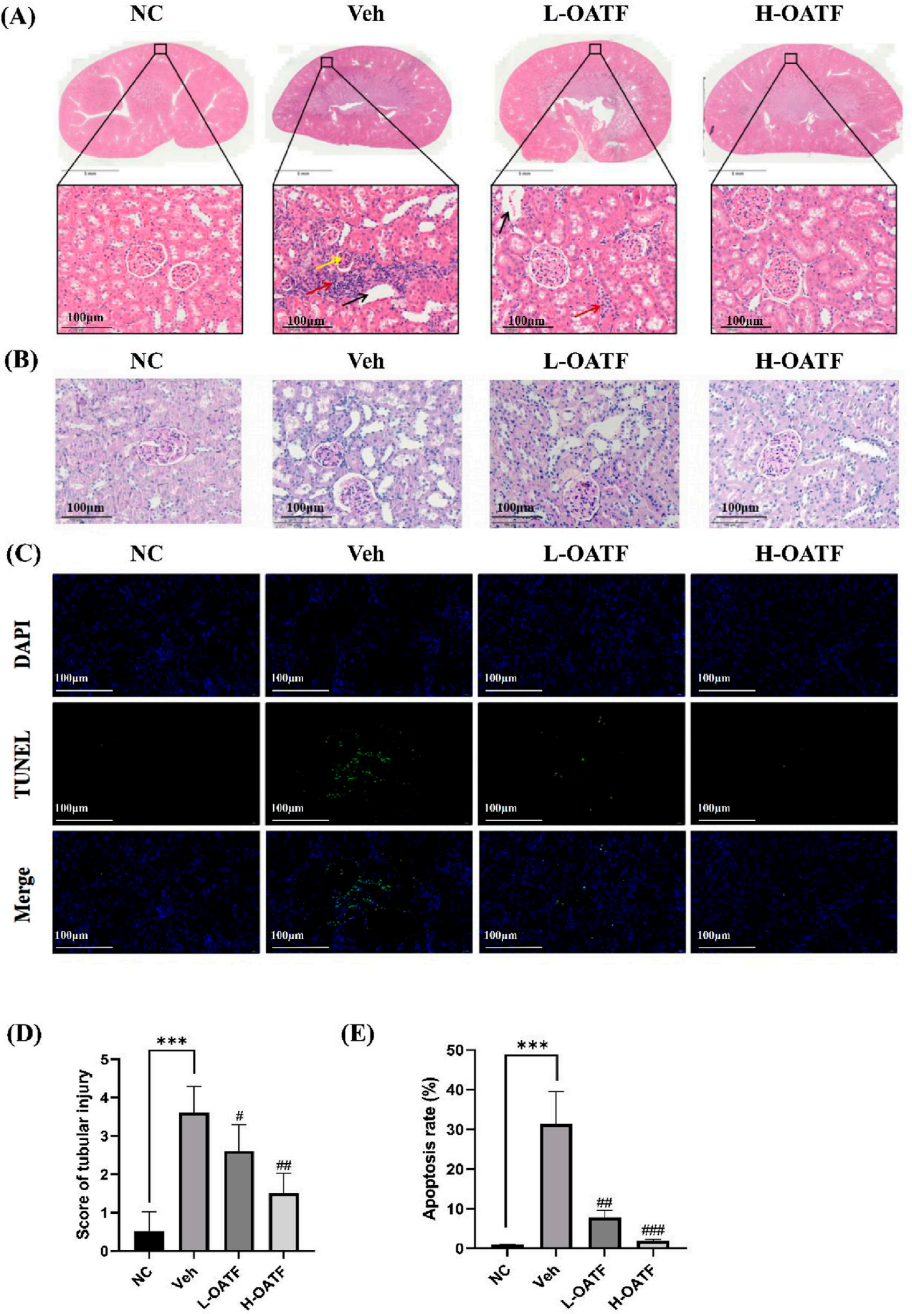
OATF alleviates renal pathological changes and apoptosis in EG + AC-induced kidney injury in rats. **(A)** Representative HE-stained kidney sections showing histopathological changes. Scale bars: 5 mm and 100 μm. Yellow arrows indicate increased glomerular mesangial cells and extracellular matrix (ECM), red arrows indicate inflammatory cell infiltration, and black arrows indicate tubular dilatation. **(B)** Representative PAS-stained kidney sections showing tubular injury. Scale bars: 100 μm. **(C)** TUNEL staining was performed to evaluate renal apoptosis. Scale bar: 100 μm. **(D)** Kidney injury scores were assessed across different groups (n = 10 fields per group). **(E)** Quantification of TUNEL-positive cells (green) relative to total cells (blue) using ImageJ. Statistical significance was determined using one-way ANOVA, and values are expressed as mean ± SD. ^***^ *p* < 0.001 vs. the NC group, ^#^ *p* < 0.05 vs. the Veh group, ^##^ *p* < 0.01 vs. the Veh group, ^###^ *p* < 0.001 vs. the Veh group.

After 4 weeks of drug administration, the renal tissues of each group were evaluated histopathologically. HE staining revealed typical features of KS in the model group, including severe dilatation of renal tubules with markedly enlarged lumens, numerous epithelial cell nuclei that were consolidated and darkly stained, glomerular mesangial matrix hyperplasia, and inflammatory infiltration in the renal interstitium (Figure 7A). As shown in Figure 7B, PAS staining was used to evaluate the degree of tubular damage. Severe tubular injury was observed in the model group, whereas treatment with different doses of OATF reversed these pathological changes. This included a significant reduction in tubular damage compared with the model group, with the extent of improvement positively correlated with the treatment dose (Figure 7D). These findings suggest that OATF has the potential to attenuate renal pathological changes in rats with KS.

Given that kidney stone formation is associated with tubular injury and apoptosis (Sun et al., 2020; Ye et al., 2021; Yu et al., 2021), we further examined renal cell apoptosis using TUNEL staining. The model group exhibited a significantly higher number of TUNEL-positive cells compared to the NC group, indicating extensive apoptosis (Figures 7C,E). However, OATF treatment markedly reduced the number of apoptotic cells, suggesting its protective role against ethylene glycol and ammonium chloride-induced kidney injury.

These findings collectively indicate that OATF alleviates renal pathological changes and attenuates apoptosis in rats with kidney stones, highlighting its therapeutic potential in preventing stone-induced renal damage.

### 3.8 Observation of EGFR, p-PI3K/PI3K and p-AKT1/AKT1protein expressions in kidney tissues by western blotting

**FIGURE 8:**
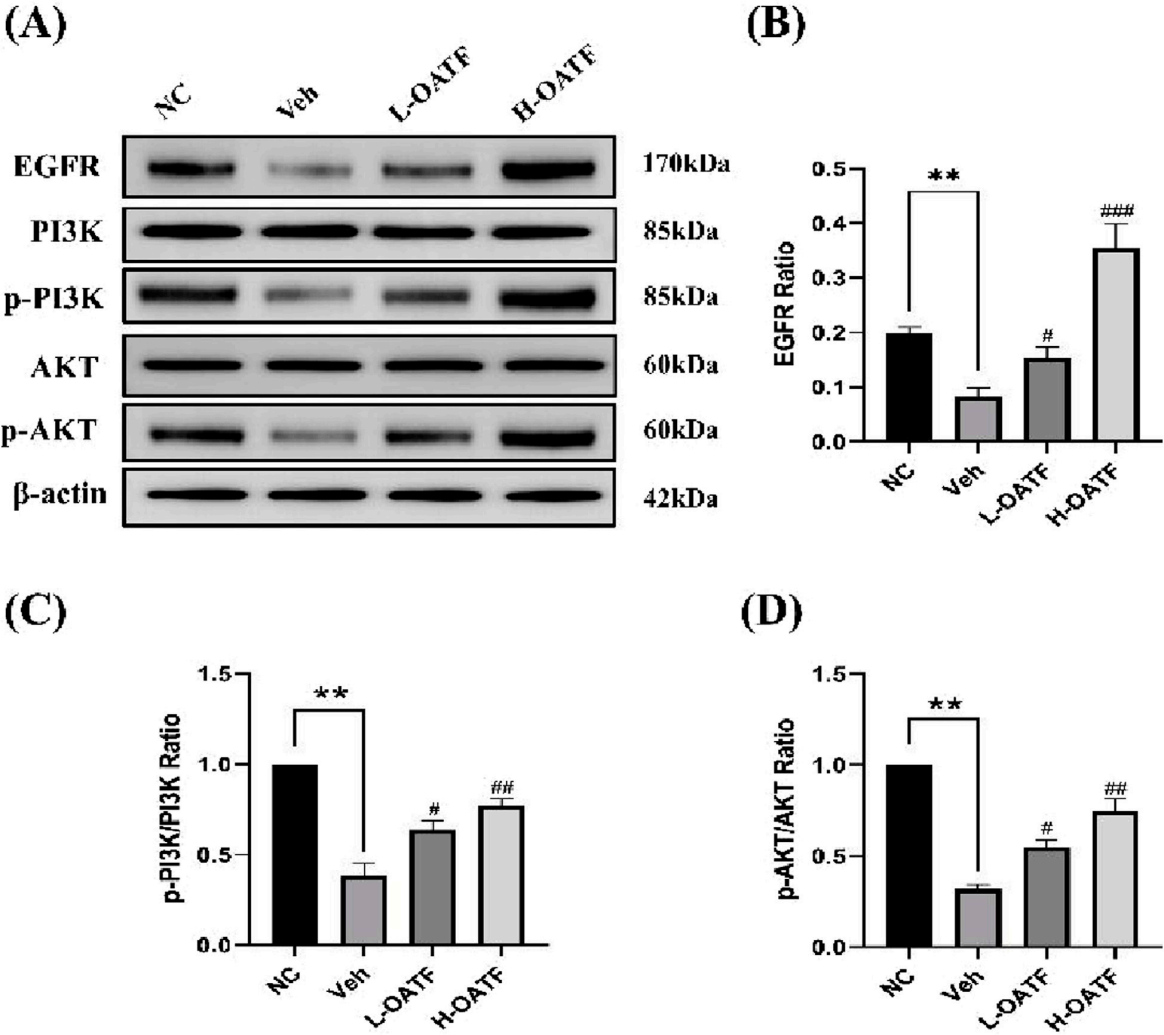
Observation of EGFR, p-PI3K/PI3K and p-AKT1/AKT1protein expressions in kidney tissues by Western blotting. **(A–D)** Western blot analysis of EGFR, PI3K/p-PI3K, and AKT/p-AKT expression in kidney tissues across experimental groups. **p < 0.01 vs. the NC group, ^#^ *p* < 0.05 vs. the Veh group, ^##^ *p* < 0.01 vs. the Veh group, ^###^ *p* < 0.001 vs. the Veh group.

Based on network pharmacology and molecular docking results, the epidermal growth factor receptor (EGFR)/PI3K/AKT signaling pathway was selected for validation. The effects of OATF on EGFR, p-PI3K/PI3K, and p-AKT/AKT protein expression were assessed using Western blot analysis. The experimental results indicated that the levels of EGFR, p-PI3K/PI3K, and p-AKT/AKT proteins were significantly reduced in the kidney tissues of rats in the model group compared to the NC group (*P* < 0.05 or *P* < 0.01, Figures 8A,D). In contrast, the expression of these proteins was significantly upregulated in the OATF-treated group compared to the model group. These findings suggest that OATF may improve kidney function in SD rats by activating the EGFR/PI3K/AKT signaling pathway.

## 4 Discussion

**FIGURE 9:**
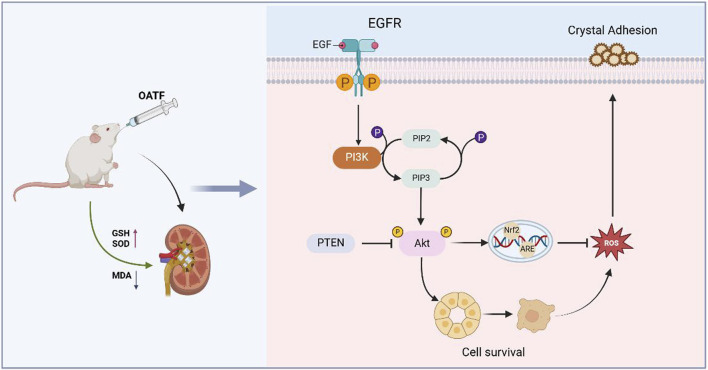
Mechanism diagram of intervention of OATF on KS. OATF exerts a protective role by activating the EGFR/PI3K/AKT pathway, which regulates cell growth and survival, thereby mitigating renal injury induced by CaOx crystal deposition.

Kidney stones disease is a common and prevalent condition in the urinary system, with incidence rates increasing annually (Khan et al., 2021). Despite significant advances in understanding the mechanisms of crystallization and stone formation, effective treatments remain limited due to slow progress in clarifying the pathophysiological mechanisms underlying stone formation. Therefore, kidney stones warrant increased attention. The development of Traditional Chinese Medicine (TCM)-based treatment modalities for kidney stones offers a potential solution to the challenges of intractability and recurrence associated with this condition. Network pharmacology has been widely applied to elucidate the complex therapeutic mechanisms of drugs, among which the renal tea extract OATF has garnered particular interest as a key therapeutic candidate for kidney stone treatment. Kidney tea is widely distributed in Guangdong, Yunnan, and Fujian, China (Fundamentals of Zhuang Medicine Nanning, (2019) Nanning: Guangxi Science and Technology Press, 2019), and is also cultivated in Southeast Asia and Australia (Zhou et al., 2024b). The Dai medical texts *Bei Ye Jing* and *Dang Ha Ya* document that it has been used for the treatment of urinary disorders and rheumatoid arthritis for over 2,000 years (Ashraf et al., 2018). On the other hand, this time-honored medicine is widely used in the treatment of kidney inflammation, kidney stones, and urinary difficulties. It is also one of the most popular medicinal plants in Thai traditional medicine for managing urinary disorders (Zhou et al., 2024b). Natural flavonoids, a major bioactive component of renal tea, have received widespread attention for their strong antioxidant, anti-inflammatory, anti-apoptotic, and immunomodulatory properties (Li et al., 2023; Zhu et al., 2024). However, the specific role of OATF in the treatment of kidney stone disease remains unclear. In this study, we predicted the therapeutic potential of OATF for kidney stones through network pharmacology and confirmed its efficacy via *ex vivo* experiments, validating the targets and signaling pathways identified by network analysis. Our results revealed a strong correlation between OATF and apoptotic targets in kidney stones. Enrichment analysis of the constructed PPI network indicated that the EGFR/PI3K/AKT pathway may be linked to the anti-apoptotic effect of OATF. PI3K is a lipid kinase with important functions in both normal and aberrant cellular processes, and its activation of protein kinase B (PKB or AKT) regulates cell proliferation, differentiation, apoptosis, and migration. Studies have shown that the PI3K/AKT pathway plays a critical role in inflammation, oxidative stress, and apoptosis in renal diseases and is closely associated with kidney stones (Chen et al., 2020; Yuan et al., 2021). Our experiments demonstrated that OATF exerts pharmacological effects through the PI3K/AKT pathway, and we subsequently predicted the binding affinity of OATF to core targets using molecular docking analysis. We hypothesized that OATF might attenuate calcium oxalate (CaOx)-induced apoptosis in renal cells via the EGFR/PI3K/AKT pathway, and this hypothesis was supported by both *in vivo* and *ex vivo* experiments. These results not only elucidate the function and mechanism of OATF but also present a novel therapeutic target for patients with CaOx kidney stones (Figure 9).

Elevated oxalate concentration is a key trigger for calcium oxalate crystal formation (Ye et al., 2020), and supersaturated oxalate leads to renal tubular epithelial cell damage. A growing body of research suggests that the adhesion or endocytosis of crystals by renal tubular epithelial cells plays a crucial role in stone formation (Gill et al., 1982). Excess oxalate disrupts the function of enzymes that regulate phosphatidylserine asymmetry on the cell membrane, indirectly altering membrane polarity. These changes create binding sites for extracellular crystalline particles, thereby promoting the early growth of stones (Thamilselvan et al., 2012). Furthermore, damaged renal tubular epithelial cells enhance crystal adhesion, contributing to renal stone formation (Nogales et al., 2022). In this study, we observed that different concentrations of OATF significantly reduced CaOx crystal adhesion in HK-2 renal tubular epithelial cells.

Furthermore, as a key byproduct of oxidative stress, ROS directly affect the health and function of renal cells. Excess ROS can damage cell membranes, proteins, and DNA, leading to tubular epithelial cell injury, pathological calcification in the renal collecting system, the formation of Randall’s plaques, and the subsequent development of calcium oxalate stones (He et al., 2024; Hou et al., 2024). Clinical studies have shown that patients with kidney stones have lower levels of antioxidant enzymes. Furthermore, ROS chemically modify intracellular molecules, including nucleotides, proteins, and lipids, disrupting their normal functions (El Abed et al., 2019). These modifications trigger cellular inflammation and autophagy, ultimately leading to renal tubular epithelial cell damage. SOD activity, along with MDA and GSH levels, reflect tissue oxidative status, lipid peroxidation, and cellular damage (Goc et al., 2017). By measuring MDA, GSH, SOD, and ROS levels in cells, we found that OATF significantly reduced oxidative stress levels induced by supersaturated oxalate and CaOx both *ex vivo* and *in vivo*. In addition, OATF improved renal function in SD rats, reducing serum creatinine, urea nitrogen, calcium, and phosphorus levels, and decreased calcium oxalate crystal deposition and damage to renal tubules.

Since the EGFR/PI3K/AKT pathway plays a critical role in the mechanism of action of OATF, we confirmed its activation using Western blotting. Structurally, EGFR consists of extracellular ligand-binding, transmembrane, and intracellular kinase domains. Ligand binding triggers EGFR activation through dimerization and subsequent phosphorylation of tyrosine residues, which serve as docking sites for downstream signaling molecules. This process activates key pathways, including PI3K/AKT and RAS/MAPK, which regulate cell survival and apoptosis (Beker et al., 2019; Kumagai et al., 2021). Apoptosis, oxidative stress, and inflammation are closely linked to kidney disease, with the PI3K/AKT pathway playing a particularly vital role in these processes (Wang et al., 2024). Our study confirms the anti-apoptotic effects of OATF in *ex vivo* models and identifies the EGFR/PI3K/AKT signaling pathway as a key mechanism, as predicted by network pharmacology, that may influence kidney stone formation.

## 5 Conclusion

In conclusion, this study integrates network pharmacology analysis with experimental validation to elucidate the molecular mechanism of OATF in treating KS. The findings indicate that OATF exerts its anti-KS effects through multiple bioactive components, targets, and pathways. Notably, experimental results confirmed that OATF reduces CaOx crystal deposition by modulating the EGFR/PI3K/AKT signaling pathway, highlighting its protective role in kidney stone formation. These results provide valuable insights into the pharmacological mechanism of OATF and support its potential as a promising therapeutic candidate for KS.

## Data Availability

The raw data supporting the conclusions of this article will be made available by the authors, without undue reservation.
